# Solid Pseudopapillary Neoplasm of the Pancreas Presenting With Gastric Outlet Obstruction

**DOI:** 10.7759/cureus.24092

**Published:** 2022-04-13

**Authors:** Jyotirmoy Biswas, Kankana Karpha, Siddhartha Nath, Arkadeep Dhali, Gopal Krishna Dhali

**Affiliations:** 1 Medicine, College of Medicine and Sagore Dutta Hospital, Kolkata, IND; 2 Gastrointestinal Surgery, School of Digestive and Liver Diseases, Institute of Postgraduate Medical Education and Research, Kolkata, IND; 3 Gastroenterology, School of Digestive and Liver Diseases, Institute of Postgraduate Medical Education and Research, Kolkata, IND

**Keywords:** outcome, surgery, small intestinal obstruction, cystic neoplasm of the pancreas, solid pseudopapillary neoplasm

## Abstract

Solid pseudopapillary neoplasm (SPN) of the pancreas are rare tumors accounting for a minor portion of all exocrine pancreatic tumors. It usually occurs in young women. It has a very low malignant potential with a relatively indolent clinical course. A small subset of patients exhibits pathological features of malignancy. Herein, we present a rare case of pancreatic SPN that presented with gastric outlet obstruction. Despite the characteristic computed tomography (CT) findings, due to its rarity, it was missed in more common conditions such as gastrointestinal stromal tumors. In our case, we found that the tumor was causing extrinsic duodenal compression leading to gastric outlet obstruction, creating a diagnostic dilemma.

## Introduction

Pancreatic solid pseudopapillary tumor (SPT) is a rare neoplasm, usually characterized by a well-encapsulated mass, with very low malignant potential. It is predominantly seen in young females, with a female/male ratio of 9.5:1 [1]. It was first described by Frantz in 1959 [2]. The first surgical resection of this neoplasm was performed by Grosfeld, and the electron microscopic appearance was first described by Hamoudi et al. in 1970 [3].

These are often clinically asymptomatic. Due to its indolent course and low malignant potential, mostly, it presents with gradually enlarging abdominal mass or nonspecific abdominal discomfort [4,5]. On palpation, the abdomen is usually non-tender but may become tender if obstructive symptoms occur in cases of large lesions compressing the adjacent viscera. SPT can be visualized in many imaging modalities, such as ultrasonography (USG), computed tomography (CT), and magnetic resonance imaging (MRI), which can be used to differentiate it from other pancreatic lesions [6]. Herein, we present a case of SPN in a male patient where the tumor was causing extrinsic duodenal compression, leading to gastric outlet obstruction.

## Case presentation

A 22-year-old male presented with intermittent, dull aching type of abdominal pain for the last four months. It was localized to the epigastrium and reached a peak within 45 minutes of food intake, persisting for 3-4 hours. It was non-radiating and not related to bowel habits but associated with multiple episodes of non-bilious vomiting that contained food particles. He denied any history of abdominal distension, jaundice, fever, weight loss, appetite loss, blood in stool, and altered bowel and bladder habits. There was no history of passage of clay-colored stool or biliary instrumentation. He also gave a history of alcohol abuse for the past three years.

Physical examination was noncontributory. Provisional diagnoses considered at this point were pyloric stricture, gastric tumor, cystic pancreatic tumor, and duodenal mass.

Biochemical and serological investigations are shown in Table 1. Blood cell counts and renal function tests were within normal limits. Inflammatory markers such as C-reactive protein and pro-calcitonin were within the normal range.

**Table 1:** Details of the initial laboratory investigations NR: nonreactive

Laboratory test	Laboratory value (normal range)
Total bilirubin	0.7 mg/dL (0.2-1.2 mg/dL)
Aspartate transaminase	85 U/L (8-48 U/L)
Alanine transaminase	75 U/L (7-55 U/L)
Alkaline phosphatase	230 IU/L (44-147 IU/L)
Albumin	2.2 g/dL (3.5-5.5 g/dL)
Amylase	52 U/L (40-140 U/L)
Lipase	88 U/L (0-160 U/L)
Hepatitis B surface antigen	NR
Anti-hepatitis C virus antibody	NR
HIV serology	NR
Autoimmune hepatitis markers	NR

**Figure 1:**
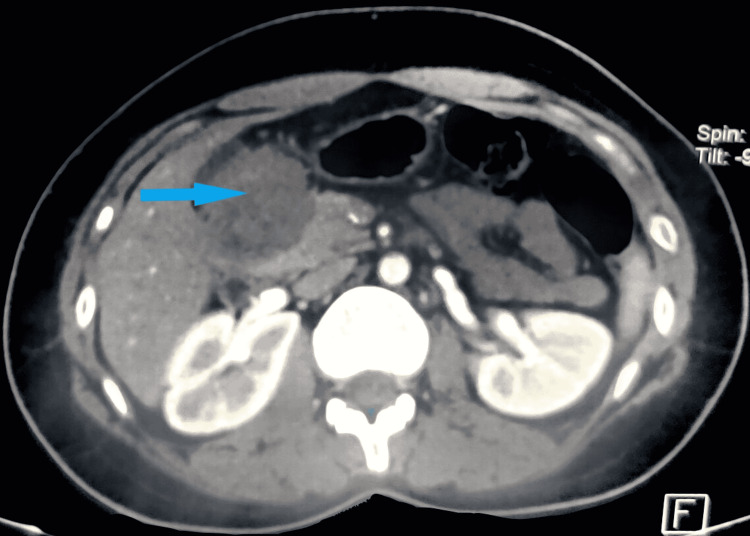
Contrast-enhanced computed tomography of the abdomen showing a well-defined mild heterogeneously enhancing soft tissue density measuring about 5.8 × 4 cm noted in the right subhepatic space around the pyloroduodenal junction (blue arrow)

Transabdominal ultrasound evaluation of the abdomen showed a homogeneous hypoechoic soft tissue texture lesion adjacent to the gallbladder fossa, duodenum, and pancreas measuring 4.4 × 4.8 × 4 cm with no internal calcifications. Contrast-enhanced computed tomography of the abdomen showed a well-defined mild heterogeneously enhancing soft tissue lesion measuring about 5.8 × 4 cm noted in the right subhepatic space around the pyloroduodenal junction, suggestive of gastrointestinal stromal tumor (Figure 1). Upper gastrointestinal endoscopy showed extrinsic compression at the D1-D2 junction. Carbohydrate antigen 19-9 was 15 U/mL (normal range: 0-37 U/mL).

**Figure 2:**
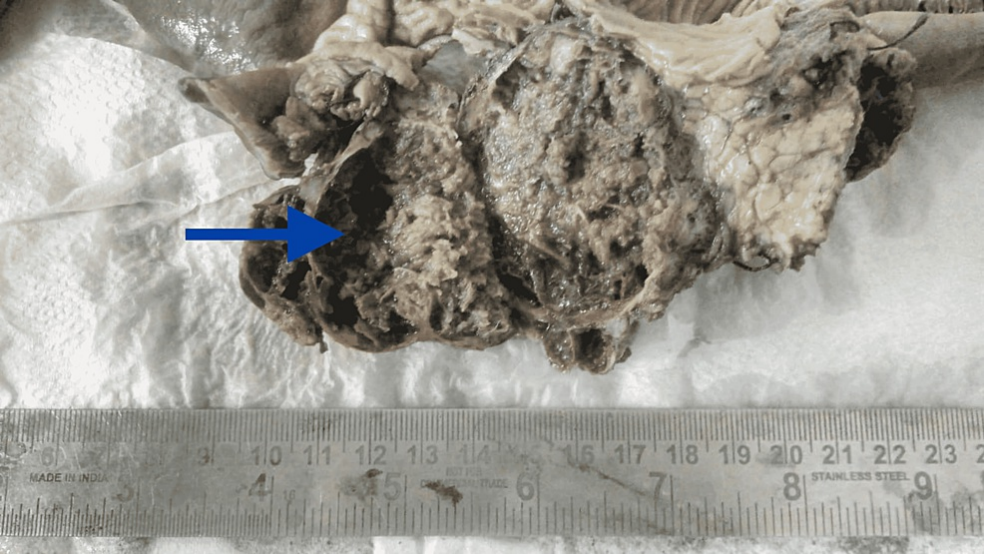
Gross specimen post-Whipple’s procedure showing the 6 × 4 cm space-occupying lesion of the pancreatic head (blue arrow)

**Figure 3:**
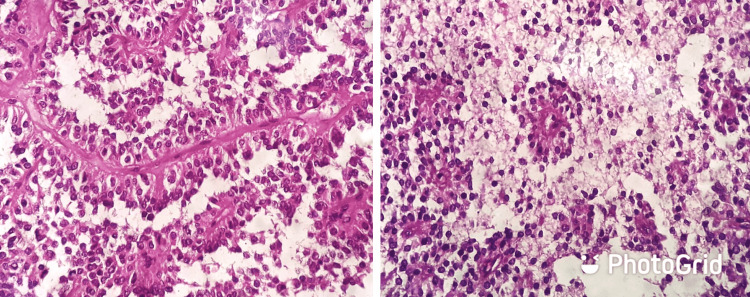
Hematoxylin and eosin image showing solid and cystic areas with dyscohesive, round monomorphic cells forming pseudopapillae around hyalinized stroma suggesting pseudopapillary tumor

The differential diagnoses considered were exophytic gastrointestinal stromal tumor and exophytic pancreatic tumor. In view of the above findings, the patient was taken up for Whipple’s pancreaticoduodenectomy and feeding jejunostomy under general anesthesia. Intraoperatively, a 6 × 4 cm space-occupying lesion of the pancreatic head occupying the pancreaticoduodenal groove was observed (Figure 2). The resected specimens were sent for histopathological examination, which revealed a cellular neoplasm consisting of sheets of small, uniform tumor cells surrounding delicate hyalinized fibrovascular stroma forming pseudopapillae. Intracellular and extracellular hyaline globules along with foamy cells were also seen. The overall histological features were suggestive of a solid pseudopapillary tumor (Figure 3). The tumor margin was negative for malignant cells, and there was no breach in the capsule of the lesion. The resected lymph nodes were free from malignant cells. The patient had an uneventful postoperative recovery and was discharged on postoperative day 7. A lifelong need for insulin and pancreatic enzyme supplementation was explained. The patient was advised six-monthly follow-up with ultrasonographic imaging of the abdomen and was doing well on the 12-month follow-up. There was no radiological evidence of recurrence of the disease on check ultrasonographic imaging.

## Discussion

Solid pseudopapillary neoplasm (SPN) is described as a rare pancreatic epithelial tumor that accounts for 1%-2% of all non-endocrine pancreatic tumors. There has been a steady increase in the number of diagnosed cases of cystic neoplasm of the pancreas including SPN. This is mostly attributed to the increased use of various radiological investigations [7]. It is frequently observed in young people and women, with a female/male ratio of 9.5:1 [1,6]. Although the cause of marked female predominance is still unknown, Kosmahl et al. have suggested that the migration of primordial ovarian cells to the developing pancreatic tail may give rise to SPN [8]. Recent studies have identified various molecular events involved in the pathogenesis of SPN. There is a clear distinction in the genetic profile of SPN from that of pancreatic adenocarcinoma. SPNs show β-catenin gene mutations, which is a downstream regulator of Wnt signaling [9,10].

The World Health Organization has classified SPN as a low-grade malignant tumor of the exocrine pancreas [11]. It is usually noninvasive in nature and hence asymptomatic and only detected after growing as large as 2.5-10 cm in diameter. Clinical manifestations are therefore due to the compression of the adjacent structures. These are equally distributed in the head, neck, body, and tail of the pancreas. A few cases of SPN of the pancreas associated with sinistral portal hypertension were previously reported [12]. There is also a case of SPN presenting with obstructive jaundice [3]. Gastric outlet obstruction as a presenting feature of SPN has been rarely reported before [13]. The majority of tumors are diagnosed through radiological modalities such as ultrasound or computed tomography of the abdomen, but magnetic resonance imaging helps in defining the hypervascular, well-encapsulated, round tumors with mixed cystic and solid components. Endoscopic ultrasonography-guided fine-needle aspiration biopsy is beneficial for preoperative pathological diagnosis [14]. Certain histopathological features, such as extensive necrosis, nuclear atypia, high mitotic rate, sarcomatoid areas, and immunohistochemistry evaluation showing high expression of Ki-67, suggest the aggressive behavior of the tumor [15-17].

Despite reports suggesting locally aggressive features, the tumor has a very low-grade malignant potential and tends to have a favorable prognosis, even in the presence of metastatic disease. The overall five-year survival is as high as 97% in patients undergoing surgical resection [18].

## Conclusions

In this case, the solid pseudopapillary neoplasm (SPN) of the pancreas was affecting a male patient. Herein, we report that SPN can cause complications such as gastric outlet obstruction despite its indolent course. Moreover, in a background of alcohol abuse with a pancreatic head mass, clinicians may face a diagnostic dilemma to rule out malignancy versus groove pancreatitis. Timely resection on diagnosis provides long-term survival. Histopathological evaluation of the sample stays as the investigation of choice as every pancreatic neoplasm behaves uniquely.
